# Hyperpigmented spots at fundus examination: a new ocular sign in Neurofibromatosis Type I

**DOI:** 10.1186/s13023-021-01773-w

**Published:** 2021-03-23

**Authors:** Antonietta Moramarco, Fabiana Mallone, Marta Sacchetti, Luca Lucchino, Emanuele Miraglia, Vincenzo Roberti, Alessandro Lambiase, Sandra Giustini

**Affiliations:** 1 Department of Sense Organs, Sapienza University of Rome, Policlinico Umberto I, Viale del Policlinico 155, 00161 Rome, Italy; 2 Department of Dermatology and Venereology, Sapienza University of Rome, Policlinico Umberto I, Rome, Italy

**Keywords:** Neurofibromatosis Type I (NF1), Hyperpigmented spots (HSs), Rare diseases, Choroidal nodules, Ultra-wide field (UWF), Near-infrared reflectance (NIR) imaging OCT (NIR-OCT), Enhanced depth imaging OCT (EDI-OCT), Indirect ophthalmoscopy

## Abstract

**Background:**

Neurofibromatosis Type I (NF1), also termed von Recklinghausen disease, is a rare genetic disorder that is transmitted by autosomal dominant inheritance, with complete penetrance and variable expressivity. It is caused by mutation in the NF1 gene on chromosome 17 encoding for neurofibromin, a protein with oncosuppressive activity, and it is 50% sporadic or inherited. The disease is characterized by a broad spectrum of clinical manifestations, mainly involving the nervous system, the eye and skin, and a predisposition to develop multiple benign and malignant neoplasms. Ocular diagnostic hallmarks of NF1 include optic gliomas, iris Lisch nodules, orbital and eyelid neurofibromas, eyelid café-au-lait spots. Choroidal nodules and microvascular abnormalities have recently been identified as additional NF1-related ocular manifestations. The present study was designed to describe the features and clinical significance of a new sign related to the visual apparatus in NF-1, represented by hyperpigmented spots (HSs) of the fundus oculi.

**Results:**

HSs were detected in 60 (24.1%) out of 249 patients with NF1, with a positive predictive value of 100% and a negative predictive value of 44.2%. None of the healthy subjects (150 subjects) showed the presence of HSs. HSs were visible under indirect ophthalmoscopy, ultra-wide field (UWF) pseudocolor imaging and red-only laser image, near-infrared reflectance (NIR)-OCT, but they were not appreciable on UWF green reflectance. The location and features of pigmentary lesions matched with the already studied NF1-related choroidal nodules. No significant difference was found between the group of patients (n = 60) with ocular HSs and the group of patients (n = 189) without ocular pigmented spots in terms of age, gender or severity grading of the disease. A statistically significant association was demonstrated between the presence of HSs and neurofibromas (*p* = 0.047), and between the presence of HSs and NF1-related retinal microvascular abnormalities (*p* = 0.017).

**Conclusions:**

We described a new ocular sign represented by HSs of the fundus in NF1. The presence of HSs was not a negative prognostic factor of the disease. Following multimodal imaging, we demonstrated that HSs and choroidal nodules were consistent with the same type of lesion, and simple indirect ophthalmoscopy allowed for screening of HSs in NF1.

## Background

Neurofibromatosis type 1 (NF1), formerly called von Recklinghausen disease, is an autosomal dominant condition caused by a mutation in the NF1 gene located on chromosome 17q11.2 encoding for neurofibromin, a 2818 aminoacids protein which functions as a negative regulator of RAS activity. In about 50% of individuals, the disease is caused by spontaneous mutation and in the other 50%, the disease is inherited [1]. NF1 affects approximately 1:2500–1:3500 individuals worldwide, regardless of gender and ethnicity, with a penetrance of about 100% [2, 3]. It presents with significant clinical heterogeneity and age-related nature of clinical expression [4]. NF1 may involve nearly every organ system in the body, with significant inter-familial and intra-familial variability.

It is characterized by the appearance of various cutaneous, ocular and neurological manifestations, and an increased susceptibility to develop tumors. The disease may also present as mosaicism, also called segmental NF1. In these cases, NF1 maintains the usual characteristics of the disease but is localized only in a body segment [4].

NF1 pathogenesis involves neural crest-derived melanocytes, Schwann cells, prevertebral ganglion and sympathetic neurons [5, 6]. The eye and ocular adnexa are commonly involved in NF1 [7]. Some ocular manifestations of NF1, including optic gliomas [8], iris Lisch nodules [9], orbital and eyelid neurofibromas, eyelid café-au-lait spots, are diagnostic hallmarks of the disease. Other ocular features have recently been characterized and are not currently diagnostic for NF1, including choroidal nodules [10] and retinal microvascular abnormalities [11–13].

The purpose of the present study was to describe a new ocular feature, represented by hyperpigmented spots (HSs) at fundus examination in a large sample of NF1 patients and control subjects, along with associations with demographical and clinical parameters.

The morphological appearance and localization of HSs at the level of chorioretinal structures were studied on ultra-wide field (UWF) images and carefully compared with Spectral Domain OCT (SD-OCT) scans and ophthalmoscopic findings.

In addition, it was performed a subgroup analysis between NF1 patients with and without HSs to evaluate differences in terms of age, gender, severity grading of pathology, frequency of the NIH diagnostic criteria, NF1-related choroidal nodules and retinal microvascular abnormalities.

Sensitivity and specificity of HSs compared with standard NIH criteria were also evaluated.

To our knowledge, it is the first article reporting this ocular manifestation in NF1.

## Methods

In the present observational, cross sectional study, two hundred forty-nine patients with diagnosis of NF1 and 150 healthy control subjects matched for age, gender and race, were consecutively evaluated for the presence of HSs at fundus examination between October 2015 and December 2019 at the University of Rome ‘Sapienza’, Umberto I Hospital, Italy. All of the procedures conformed to the tenets of the World Medical Association Declaration of Helsinki. A written informed consent was obtained from all subjects and from parents in case of minor age. These consecutive patients were 139 females and 110 males, between 5 and 78 years of age (mean age: 33 ± 17.1 years), whereas the control group was composed of 88 females and 62 males, mean age: 34.5 ± 16.9 years (age from 6 to 80 years). All patients and control subjects were Caucasians.

We included all patients diagnosed with NF1 based on the National Institutes of Health (NIH) criteria [14]. Each patient underwent dermatologic and ophthalmologic examination. The severity grading was evaluated according to the Riccardi Scale [15, 16].

Exclusion criteria were: non-explorable fundus because of media opacities, myopia greater than 6D (Spherical Equivalent), any other previous or coexisting ocular disease that could affect fundus aspect.

Healthy subjects were recruited from outpatients of the eye clinic according to the exclusion criteria.

All patients underwent detailed ophthalmological examination, including: Snellen best-corrected visual acuity (BCVA) measurement, anterior segment slit-lamp examination, Goldmann applanation tonometry, mydriatic indirect fundus biomicroscopy, ultra-wide field (UWF) scanning laser ophthalmoscopic imaging (Optos 200Tx™ imaging system, Optos PLC, Dunfermline, Scotland, UK), near infrared reflectance (NIR) retinography by using the spectral domain optical coherence tomography (SD-OCT) and cross-sectional SD-OCT in enhanced depth imaging (EDI) modality. Indirect ophthalmoscopy allowed for detection of HSs of the fundus oculi, whereas SD-OCT and UWF images were performed in order to evaluate the extension and localization of HSs through different wavelenghts.

SD-OCT images were obtained with the Spectralis OCT (Spectralis Family Acquisition Module, V 5.1.6.0; Heidelberg Engineering, Heidelberg, Germany), following a standardized protocol.

Near-infrared (NIR) OCT (787 nm) provided better visualization of the subretinal and choroidal structures than the light of the shorter red laser wavelengths used in the Optos 200TxTM imaging system (633 nm). All participants were imaged with pseudocolor, simulated white light (488–633 nm) and green light (GAF) autofluorescence (532 nm) on UWF imaging [17].

Furthermore, enhanced depth imaging was used to improve the visualization of the choroid as described by Spaide et al. [18]. Specifically, cross sectional SD-OCT images in EDI technique allowed precise evaluation of size, contour, level of depth and inward extension of choroidal nodules, as well as the status of the surrounding tissues including choriocapillaris, medium and large size choroidal vessels, RPE/Bruch's membrane and retina.

Two, independent ophthalmologists carefully assessed the presence of HSs under fundus examination and by the use of instrumental exams, considering positively only subjects with affirmative judgment by both the experts. The assessors were masked to whether or not the patients and controls had NF1.

The normality of data was assessed by Shapiro–Wilk test. Descriptive statistical analysis was performed in all collected data. Continuous variables were presented as mean ± standard deviation (SD), and the independent Student's t-Test was used to compare subgroups of patients. Categorical variables were expressed as frequencies and percentages and Fisher’s exact test was used for comparison analysis.

A subgroup comparison analysis between NF1 patients with and without HSs was conducted in order to evaluate the associations with demographical and clinical parametres.

To evaluate the predictability and the diagnostic accuracy of the HSs in comparison with the standard NIH diagnostic criteria, we calculated the sensitivity and specificity with corresponding 95% confidence intervals. Intraobserver and interobserver agreement was evaluated with the K Cohen Coefficient. A value of *p* ≤ 0.05 was considered statistically significant. Statistical analysis was performed using Graph Pad vers. 8.0.2 and IBM® SPSS® Statistics version 24.0 (IBM Corp., Armonk, NY, USA) on the Windows 10 Home edition platform. v22 (IBM SPSS Statistics, IBM®, IL, USA).

## Results

**Fig. 1:**
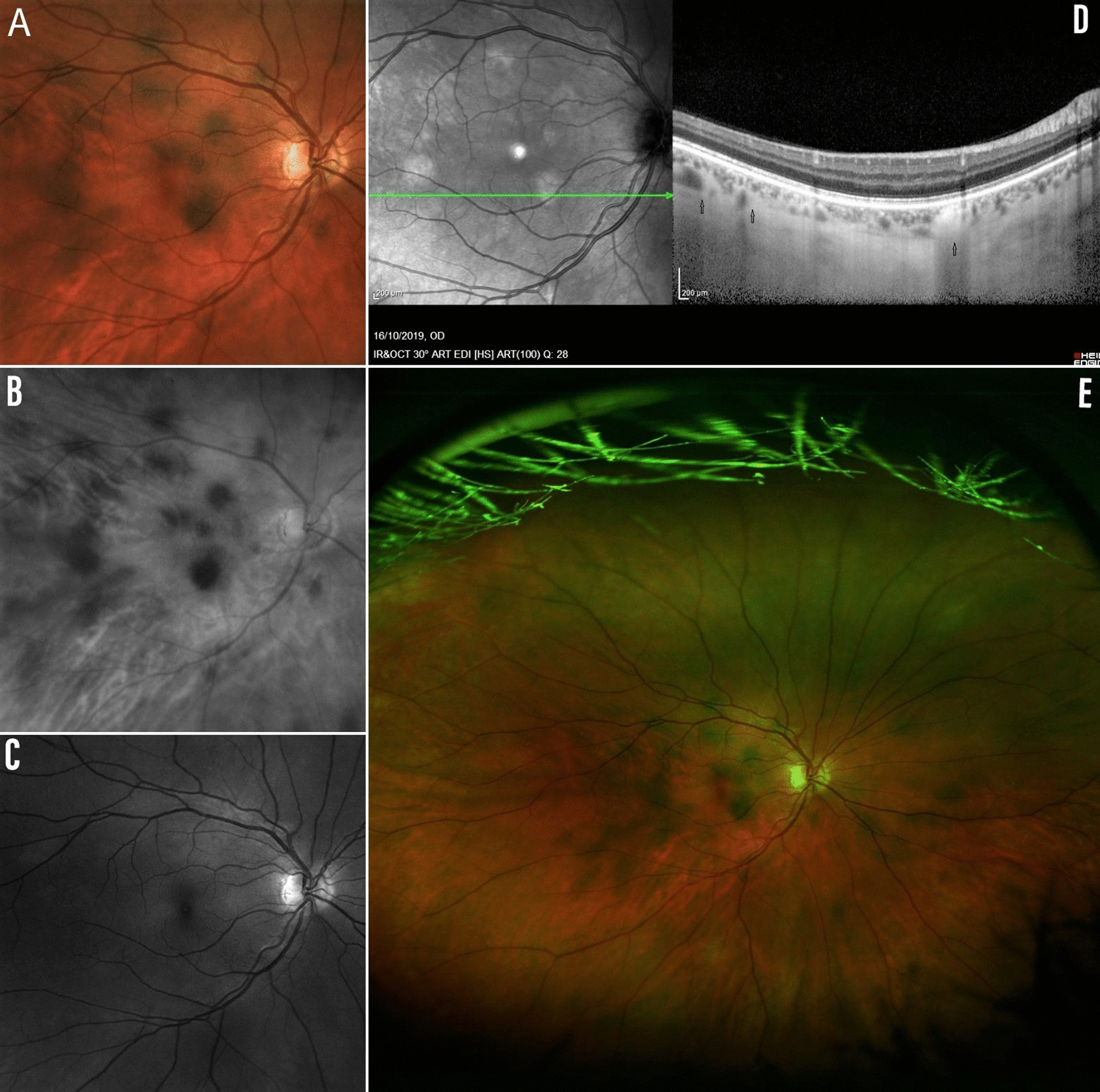
**a** HSs on UWF simulated white light-only image. **b** HSs on UWF red-only laser image. **c** UWF green-only laser image. No spots detection. **d** NIR-OCT image showing hyperreflective choroidal nodules, and cross-sectional SD-OCT in EDI modality showing the different inward extension of choroidal nodules (black arrows). **e** HSs on UWF pseudocolor composite image (simulated white light, green laser, red laser)

**Fig. 2:**
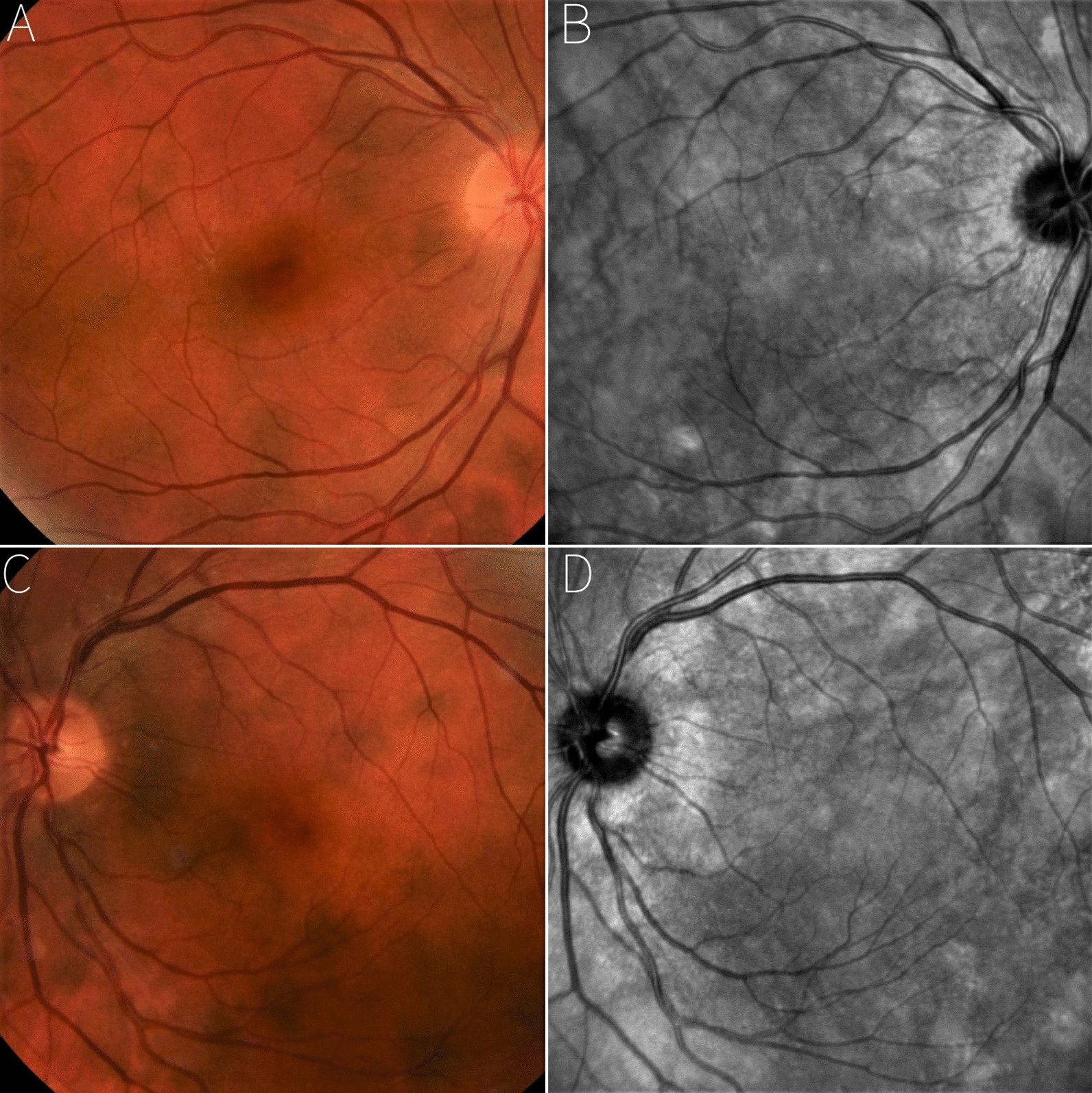
Matching between HSs of the fundus and hyperreflective areas on infrared light: comparison analysis between UWF simulated white light-only image (**a**) Vs NIR-OCT image (**b**)

Demographic and clinical characteristics of the 249 patients diagnosed with NF-1 and controls (150 patients) are listed in Table 1. In Table 2, it is reported the frequency of the NIH diagnostic criteria, NF-1-related choroidal nodules, retinal microvascular abnormalities and HSs between patients and controls. We observed the presence of HSs at fundus examination in 60 (24.1%) out of 249 patients diagnosed with NF1 (Table 1), whereas no HSs were observed within our control group. In all patients, HSs were predominantly distributed to the posterior pole. Among the 60 patients with HSs, 50 showed bilateral involvement and 10 were monolateral. Interestingly, no significant difference was found between the group of patients showing HSs and the group of patients (n = 189) without ocular HSs in terms of age, gender or severity grading of NF1 (Table 3). Among the NIH diagnostic criteria, we found a statistically significant association between the presence of HSs and neurofibromas (*p* = 0.047). In addition, we observed a statistically significant association between the presence of HSs and NF1-related retinal microvascular abnormalities (*p* = 0.017) (Table 3). The diagnostic indicators of the HSs and each of the standard NIH diagnostic criteria are compared in Table 4. The diagnostic sensitivity of the HSs was 24.1% (CI 19.2 to 29.8), whereas the specificity was 100% (CI 97.5 to 100). The intraobserver and interobserver agreement was excellent in both the ophthalmoscopic and UWF-based assessment. (Kappa = 0.874). The calculated values apply specifically to the present study sample. The HSs were detectable on indirect ophthalmoscopy, UWF pseudocolor and red laser imaging. On the other hand, they were not appreciable under green laser on UWF retinal imaging (Fig. 1a–c, e). While comparing instrumental exams, we observed that HSs of the fundus matched with hyperreflective areas on NIR-OCT, attributable to the previously described choroidal NF1 nodules, in terms of size and location (Fig. 2). Cross-sectional SD-OCT in EDI modality allowed us to properly identify different levels of extension of choroidal nodules from the deep towards the inner choroid. Specifically, we observed that choroidal nodules with the most inward extension on cross sectional OCT resulted to be detectable as HSs on UWF pseudocolor images as well as hyperreflective areas on NIR-OCT (Fig. 1d).

**Table 1:** Demographic and clinical characteristics of the 249 patients with NF1 and 150 control subjects

Characteristics	NF1 (n = 249)	Control (n = 150)	*P* value
Age (mean ± SD)	33 ± 17.1	34.5 ± 16.9	0.407^b^
Gender (n)			
Males	110	62	0.603^a^
Females	139	88	
Refractive error (%)			
Myopia	21.3	22.7	0.823^a^
Hyperopia	6.4	9.3	0.424^a^
Astigmatism	25.7	20	0.223^a^

^a^Fisher's Exact Test

^b^Student’s t-test for independent samples

**Table 2:** Frequency of the NIH diagnostic criteria, NF-1-related choroidal nodules, retinal microvascular abnormalities and HSs between patients and controls

NIH Diagnostic Criteria (%)	NF1 patients (%) (n = 249)	Controls (%) (n = 150)	*P* value
Café-au-lait spots	98.1	0	< 0.001^a^
Freckling	91.3	0	< 0.001^a^
Lisch nodules	88	0	< 0.001^a^
Neurofibromas	77.5	0	< 0.001^a^
Optic pathway gliomas	9.6	0	< 0.001^a^
Distinctive osseous lesions	2.5	0	< 0.001^a^
First-degree relative affected	44.5	2.7	< 0.001^a^
Choroidal nodules^b^	95.6	0	< 0.001^a^
Retinal Microvascular Abnormalities^b^	32.1	0	< 0.001^a^
Hyperpigmented Spots^b^	24.1	0	< 0.001^a^

^a^Fisher's Exact Test

^b^Currently not included in NIH Diagnostic Criteria

**Table 3:** Subgroup comparison analysis between NF1 patients with and without HSs

Characteristics	NF1 (n = 249)	NF1 Without HS (n = 189)	NF1 with HS (n = 60)	*P* value
Age (Mean ± SD)	33 ± 17.1	31.9 ± 17.4	36.8 ± 15.5	ns^b^
Gender (n)				
Males	110	79	31	ns^a^
Females	139	110	29	
NIH diagnostic Criteria (%)				
Café-au-lait spots	98.1	97.8	100	ns^a^
Freckling	91.3	92.1	85.7	ns^a^
Lisch nodules	88	86.2	93.3	ns^a^
Neurofibromas	77.5	74.8	95.2	0.047^a^
Optic pathway gliomas	9.6	9.5	10	ns^a^
Distinctive osseous lesions	2.5	2.2	4.8	ns^a^
First-degree relative affected	44.5	46.3	33.3	ns^a^
Choroidal nodules^c^	95.6	94.7	98.3	ns^a^
Retinal Microvascular Abnormalities^c^	32.1	28	45	0.017^a^
NF1 severity grading (%)				
1	15.8	18.1	3.3	ns^a^
2	58.4	57.5	63.3	ns^a^
3	13.2	12.5	16.7	ns^a^
4	12.6	11.9	16.7	ns^a^

^a^Fisher's Exact Test

^b^Student’s t-test for independent samples

^c^Currently not included in NIH Diagnostic Criteria

**Table 4:** Comparison between HS and standard NIH diagnostic criteria: diagnostic indicators, rate (95% CI)

NIH diagnostic criteria	Sensitivity (% and 95% CI)	Specificity (% and 95% CI)
Café-au-lait spots	98.1 (94.7–99.5)	100 (97.5–100)
Freckling	97.3 (93.3–98.9)	100 (97.5–100)
Lisch nodules	87.9 (88.3–91.4)	100 (97.5–100)
Neurofibromas	77.5 (70.4–83.2)	100 (97.5–100)
Optic pathway gliomas	9.7 (6.6–14.0)	100 (97.5–100)
Distinctive osseous lesions	2.5 (0.9–6.2)	100 (97.5–100)
First-degree relative affected	44.5 (36.9–52.3)	100 (97.5–100)
Hyperpigmented spots	24.1 (19.2–29.8)	100 (97.5–100)

CI confidence interval

## Discussion

In this study, we described the presence at fundus examination of HSs, consisting of rounded, hyperpigmented areas of variable size and number with blurred margins, in 24.1% out of 249 patients diagnosed with NF1. Of note, HSs were not detectable within the control group.

Interestingly, no significant difference was observed between the group of patients with ocular HSs and the group of patients without HSs in terms of age, gender or severity grading of the disease.

Specifically, the absence of significant correlation between the severity of NF1 and the presence of HSs suggests that they are not a negative prognostic factor of the disease.

After comparative analysis, we noticed that pigmentary lesions corresponded in location, size and morphology to lesions previously described as NF1-related choroidal nodules.

Choroidal nodules are known as ovoid bodies consisting of proliferating Schwann cells arranged in concentric rings around an axon. Histologic studies showed hyperplastic Schwann cells, melanocytes, and ganglion cells in the ovoid bodies [19, 20]. These abnormalities appear as hypofluorescent patches in the early phase of indocyanine-green angiography and as bright patchy lesions under infrared monochromatic light; they are undetectable in conventional ophthalmoscopic examination or by means of autofluorescence (FAF) and fluorescein angiography (FA) [21, 22]. More recently, Kumar V. et al. identified hyper-flow areas on optical coherence tomography angiography (OCTA) of deep choroid corresponding to the bright patches of the choroidal nodules on NIR imaging [23].

Our results suggest that choroidal nodules, all visible on infrared light, may have different level of extension in the deeper choroid and different degree of pigmentation, reaching the level of visibility at fundus examination only in a minority of cases. In this respect, optical coherence tomography B-scan through the fovea showed choroidal nodules as having different levels of inward extension in the deep choroid. The most hyperreflective areas on infrared light matched well with the most pigmented areas on indirect ophthalmoscopy, UWF pseudocolor and red laser images, and with the maximum extent from the outer to the inner choroid on cross-sectional SD-OCT scans. This explains the different frequency of presentation of choroidal nodules and HSs in our sample, showing percentages of 95.6 versus 24.1, respectively. Furthermore, in this series, HSs were predominantly distributed to the posterior pole similar to the location of choroidal nodules, as the choroid within the main retinal vascular arcades is considerably rich in melanocytes and neural cells, from previous evidence [24]. With reference to the pathogenesis of HSs, melanocytes embryologically recognize a dual origin: the neural ectoderm, more specifically, the outer layer of the optic cup, which generates the melanoblasts that migrate in the pigmented epithelium of retina, iris and ciliary body, and the neural crest that gives rise to the melanoblasts that populate the uveal portion of the eye composed of choroid, the stroma of iris and ciliary body, in addition to the melanocytes of skin and hair [25]. The same origin of melanocytes, meningoblasts and Schwann cells from the neural crest is responsible for the occasional association between pigmented lesions of the uvea or skin, meningiomas and neurofibromas [6]. However, patients with NF1 also develop tumors that are not derived from the neural crest. For example, the optic glioma and retinal hamartomas are of astrocytic and, therefore, of neuroectodermal origin. The café-au-lait spots of the skin are the result of an increased production of melanin in the basal and spinous layers of the epidermis. The hyperpigmentation is imputable to an hyperactivity of the melanosomes and / or to an increase in the number of melanocytes [26–28]. At ocular level we could hypothesize the same pathogenic mechanism, ascribing the origin of the HSs of the fundus to an increase in pigment production and/or an increase in the proportion of melanocytes in the choroidal nodules. The interaction between light and intraocular tissues plays an important role in the interpretation of optical methods for diagnosing ocular disease. The retina is nearly transparent, however, the pigments contained in the RPE and choroid interact strongly with the imaging lights over a range of wavelengths. The most representative pigments of RPE are lipofuscin and melanin. The choroid also contains melanin, with an embryonic origin and different optical and biochemical properties from its RPE counterpart. The proliferation of melanocytes in NF1 patients causes a patchy choroidal thickening, resulting in a strong absorption and a subsequent backscattering of near-infrared light through the high content of melanin [29–31]. In our series, the contribution of the choroid melanin is definitely more relevant since HSs are visible under indirect ophthalmoscopy exam, NIR-OCT and red laser images, but not under GAF. More in detail, the green laser light that is used in FAF is markedly absorbed by the pigment epithelium and therefore may only detect alterations if this cell layer is also affected. In this context, we combined NIR-OCT and UWF scans to achieve multimodal imaging.

Previous articles described pigmentary lesions at fundus examination in NF1. However, they were confined to a report of two cases published in 1978, and a more recent single patient report, both describing retinal café au-lait macules [32, 33].

Specifically, Cotlier E. reported pigmentary changes at fundus examination, resembling cutaneous café au-lait spots, in association with retinal hamartomas [32]. The lesions were described as lightly pigmented, sinuous, not elevated and not well defined, differing considerably from those observed in our group in terms of color, morphology, number and size. In addition, the concomitant presence of retinal hamartomas may be likely to indicate an exudative nature of such lesions. Then, mild choroidal hypofluorescence was described in the areas of café-au-lait pigmentation on fluorescein angiography, whereas choroidal nodules are known to be undetectable on FA. However, it is not possible to carry out adequate comparison with our findings given the absence of further instrumental exams and the scarce technical means available at that time. More recently, Venkatesh R. et al. reported retinal café-au-lait macules in a patient with NF1 [33]. Specifically, the lesions appeared as pale, light-brown coloured and flat, hypo-autofluorescent on FAF, and hyperreflective on blue, green and infrared reflectance images. Thus, clinical and instrumental appearance of these abnormalities seems to deviate much from both the findings of Cotlier E. and the HSs identified in our series.

Intriguingly, our results showed a statistically significant association between the presence of HSs and neurofibromas (*p* = 0.047), that could possibly be related to the high content in Schwann cells and melanocytes and the same derivation from the neural crest [25, 34].

In addition, a statistically significant association was found between the presence of HSs and NF1-related retinal microvascular abnormalities (*p* = 0.017).

Previous studies investigated the relationship between choroidal nodules and overlying retinal microvascular changes in NF-1 [23, 35–37]. It was hypothesized that angiogenic factors secreted by the underlying choroidal nodules could have an effect on retinal vasculature [35]. Other Authors speculated that functional disorders of vasomotor nerve cells, which originate in the embryonal neural crest, could have a role in the development of retinal microvascular alterations in NF1 patients [36]. A recent study reported an abnormal retinal vessel along with thinning and low flow areas overlying the choroidal nodules at the level of choriocapillaris on OCTA in NF-1[23]. Thus, the nature of retinal vascular changes in NF-1 and the association with the underlying nodules remain unclear. Based on previous evidence, we speculated that HSs, corresponding to hyperpigmented and inward extended choroidal nodules, could possibly have a role in the development of retinal microvascular abnormalities.

Therefore, we believe that investigation on angiographic retinal and choroidal features in relation to the presence of HSs is firmly recommended.

In our sample, the HSs showed a diagnostic sensitivity of 24.1% with high diagnostic specificity (100%). However, given the rarity of HSs, it would be advisable to implement the study sample to reach conclusions on diagnostic accuracy.

## Conclusions

The present study allowed for identification of a new ocular sign in NF-1 represented by HSs at fundus examination, with a percentage of 24.1% in our sample. The presence of HSs was not a negative prognostic factor of the disease. A statistically significant association was demonstrated between the presence of HSs and neurofibromas, and between the presence of HSs and NF1-related retinal microvascular abnormalities. Additionally, we observed the matching between HSs of the fundus and the already studied NF1-related choroidal nodules, the latter detectable only under infrared monochromatic light. Through multimodal imaging, it appears that HSs and choroidal nodules would be attributable to the same type of lesion, and that only the most inward extensive and pigmented choroidal nodules would reach the level of visibility at fundus examination as HSs.

The visualization of HSs through indirect ophthalmoscopy is easy, fast and safe: this leads to a manageable identification and monitoring over time. In addition, UWF and SD-OCT imaging allowed for an accurate and reproducible assessment. Further longitudinal, prospective studies are required to provide additional data on this new clinical sign. HSs at fundus examination may represent a potential novel diagnostic criterion to be added to the other NF1 manifestations involving the ocular structures.

## Data Availability

The data used to support the findings of this study are available from the corresponding author upon request.

## Abbreviations

NF1: Neurofibromatosis Type I
HSs: Hyperpigmented spots
UWF: Ultra-wide field
NIR-OCT: Near-infrared reflectance (NIR) imaging OCT
EDI-OCT: Enhanced depth imaging OCT
NIH: National Institutes of Health
OCTA: Optical coherence tomography angiography

## References

[1] Jett K, Friedman JM (2010). Clinical and genetic aspects of neurofibromatosis 1. Genet. Med.

[2] Yap YS, McPherson JR, Ong CK, Rozen SG, Teh BT, Lee ASG (2014). The NF1 gene revisited -from bench to bedside. Oncotarget.

[3] Poyhonen, M. Risk of malignancy and death in neurofibromatosis. search.proquest.com [Internet]. [cited 2020 Oct 13]; Available from: http://search.proquest.com/openview/aae164438ad6e71247d95e4c78c14e10/1?pq-origsite=gscholar&cbl=42082 (1997) 9126041

[4] Vandenbroucke I, van Doorn R, Callens T, Cobben JM, Starink TM, Messiaen L (2004). Genetic and clinical mosaicism in a patient with neurofibromatosis type 1. Hum Genet.

[5] Stocker KM, Baizer L, Coston T, Sherman L, Ciment G (1995). Regulated expression of neurofibromin in migrating neural crest cells of avian embryos. J Neurobiol.

[6] Van Raamsdonk CD, Deo M (2013). Links between Schwann cells and melanocytes in development and disease. Pigment Cell Melanoma Res.

[7] Kinori M, Hodgson N, Zeid JL (2018). Ophthalmic manifestations in neurofibromatosis type 1. Surv Ophthalmol.

[8] Nebbioso M, Moramarco A, Lambiase A, Giustini S, Marenco M, Miraglia E (2020). Neurofibromatosis type 1: Ocular electrophysiological and perimetric anomalies. Eye Brain.

[9] Richetta A, Giustini S, Recupero SM, Pezza M, Carlomagno V, Amoruso G (2004). Lisch nodules of the iris in neurofibromatosis type 1. J Eur Acad Dermatology Venereol.

[10] Moramarco A, Giustini S, Nofroni I, Mallone F, Miraglia E, Iacovino C (2018). Near-infrared imaging: an in vivo, non-invasive diagnostic tool in neurofibromatosis type 1. Graefe’s Arch Clin Exp Ophthalmol.

[11] Moramarco A, Giustini S, Miraglia E, Sacchetti M (2018). SD-OCT in NIR modality to diagnose retinal microvascular abnormalities in neurofibromatosis type 1. Graefes Arch Clin Exp Ophthalmol..

[12] Moramarco A, Lambiase A, Mallone F, Miraglia E, Giustini S (2019). A characteristic type of retinal microvascular abnormalities in a patient with Neurofibromatosis type 1. Clin Ter.

[13] Moramarco A, Miraglia E, Mallone F, Roberti V, Iacovino C, Bruscolini A (2019). Retinal microvascular abnormalities in neurofibromatosis type 1. Br J Ophthalmol.

[14] Stumpf, DA. Neurofibromatosis. Conference statement, National Institute of Health development conference. Arch Neurol 1988;45:575–8. 3128965

[15] Riccardi V, Kleiner B. Neurofibromatosis: a neoplastic birth defect with two age peaks of severe problems. https://europepmc.org. Available from: https://europepmc.org/article/med/407957 (1977) 407957

[16] Ablon J. Gender response to neurofibromatosis 1. [cited 2020 Oct 13]; Available from: https://www.sciencedirect.com/science/article/pii/0277953695000763 (1996) 10.1016/0277-9536(95)00076-3 8745111

[17] Nam KT, Yun CM, Kim JT, Yang KS, Kim HJ, Kim SW (2015). Central serous chorioretinopathy fundus autofluorescence comparison with two different confocal scanning laser ophthalmoscopes. Graefe’s Arch Clin Exp Ophthalmol.

[18] Spaide R, Koizumi H, Pozzoni M. Enhanced depth imaging spectral-domain optical coherence tomography. [cited 2020 Oct 13]; Available from: https://www.sciencedirect.com/science/article/pii/S0002939408004182 (2008) 10.1016/j.ajo.2008.05.032 18639219

[19] Kurosawa A, Kurosawa H. Ovoid bodies in choroidal neurofibromatosis. [cited 2020 Oct 13]; https://jamanetwork.com/journals/jamaophthalmology/article-abstract/634404 (1982) 10.1001/archopht.1982.01030040919010 6816197

[20] Wolter JR (1965). Nerve fibrils in ovoid bodies: with neurofibromatosis of the choroid. Arch Ophthalmol.

[21] Rescaldani C, Nicolini P, Fatigati G, Bottoni F. 1998. Clinical application of digital indocyanine green angiography in choroidal neurofibromatosis. [cited 2020 Oct 13]; Available from: https://www.karger.com/Article/FullText/27287 10.1159/000027287 9486548

[22] Yasunari T, Shiraki K, Hattori H, Miki T. 2000. Frequency of choroidal abnormalities in neurofibromatosis type 1. [cited 2020 Oct 13]; Available from: https://www.sciencedirect.com/science/article/pii/S0140673600027161 10.1016/S0140-6736(00)02716-1 11041400

[23] Kumar V, Singh S. Multimodal imaging of choroidal nodules in neurofibromatosis type-1. Indian J Ophthalmol 2018;66:586. Available from: http://www.ijo.in/text.asp?2018/66/4/586/228465 10.4103/ijo.IJO_1095_17 PMC5892072 29582830

[24] Naumann GOH, Apple DJ (1986). Pathology of the eye.

[25] Ward WC, Simon JD (2007). The differing embryonic origins of retinal and uveal (iris/ciliary body and choroid) melanosomes are mirrored by their phospholipid composition. Pigment Cell Res.

[26] De Schepper S, Boucneau J, Lambert J, Messiaen L, Naeyaert JM (2005). Pigment cell-related manifestations in neurofibromatosis type 1: an overview. Pigment Cell Res.

[27] De Schepper S, Boucneau J, Vander Haeghen Y, Messiaen L, Naeyaert JM, Lambert J (2006). Café-au-lait spots in neurofibromatosis type 1 and in healthy control individuals: hyperpigmentation of a different kind?. Arch Dermatol Res..

[28] Takahashi M (1976). Studies on cafe au lait spots in neurofibromatosis and pigmented macules of nevus spilus. Tohoku J Exp Med.

[29] Delori FC, Pflibsen KP (1989). Spectral reflectance of the human ocular fundus. Appl Opt.

[30] Ayata A, Tatlipinar S, Kar T, Unal M, Ersanli D, Bilge AH (2009). Near-infrared and short-wavelength autofluorescence imaging in central serous chorioretinopathy. Br J Ophthalmol.

[31] Schmitz-Valckenberg S, Lara D, Nizari S, Normando EM, Guo L, Wegener AR (2011). Localisation and significance of in vivo near-infrared autofluorescent signal in retinal imaging. Br J Ophthalmol.

[32] Cotlier E. 1977. Café-au-lait spots of the fundus in neurofibromatosis. [cited 2020 Oct 13]; Available from: https://jamanetwork.com/journals/jamaophthalmology/article-abstract/632400 10.1001/archopht.1977.04450110084007 411462

[33] Venkatesh R, Jain K, Pereira A, Jain S, Aseem A, Mahendradas P (2019). Retinal cafe-au-lait macules: A rare retinal finding in a patient with neurofibromatosis type 1. Indian J Ophthalmol.

[34] Aquino JB, Sierra R (2018). Schwann cell precursors in health and disease. Glia.

[35] Cassiman C, Casteels I, Stalmans P, Legius E, Jacob J (2017). Optical coherence tomography angiography of retinal microvascular changes overlying choroidal nodules in neurofibromatosis type 1. Case Rep Ophthalmol.

[36] Abdolrahimzadeh S, Felli L, Piraino DC, Mollo R, Calvieri S, Recupero SM (2014). Retinal microvascular abnormalities overlying choroidal nodules in neurofibromatosis type 1. BMC Ophthalmol..

[37] Parrozzani R, Pilotto E, Clementi M, Frizziero L, Leonardi F, Convento E (2018). Retinal vascular abnormalities in a large cohort of patients affected by neurofibromatosis type: 1 a study using optical coherence tomography angiography. Retina.

